# Severe influenza A virus pneumonia complicated with *Curvularia lunata* infection: Case Report

**DOI:** 10.3389/fcimb.2023.1289235

**Published:** 2023-12-15

**Authors:** Yanqing Zhang, Haixia Li, Ling Chen, Fei Feng, Liping Liu, Qinghong Guo

**Affiliations:** ^1^ Key Laboratory of Gastrointestinal Diseases of Gansu Province, The First Clinical Medical College, Lanzhou University, Lanzhou, Gansu, China; ^2^ Emergency Intensive Care Unit, The First Clinical Medical College, Lanzhou University, Lanzhou, Gansu, China

**Keywords:** *Curvularia lunata*, invasive fungal infection, influenza A virus infection, severe pneumonia, treatment

## Abstract

Human infection with *Curvularia lunata* (*C. lunata*) is exceptionally rare. A 23-year-old female patient contracted both bacterial and *Curvularia lunata* infections during influenza A virus infection. Multiple etiological tests were performed repeatedly during hospitalization due to fluctuations in condition. On the 17th day after hospital admission, mold hyphae were discovered in the pathogen culture of the patient’s bronchoalveolar lavage fluid during one of these examinations. The patient was suspected to have a filamentous fungal infection. Consequently, we further obtained sputum samples for fungal culture, which confirmed the diagnosis of *Curvularia* infection. The patient, in this case, was in a critical condition, experiencing complications of lung abscess, pneumothorax, sepsis, and multiorgan failure. Despite prompt initiation of antifungal therapy including amphotericin B cholesteryl sulfate complex and isavuconazole upon detection of the fungal infection and concurrent administration of active organ function support treatment, the patient’s condition rapidly deteriorated due to compromised immune function. Ultimately, on the 27th day of treatment, the patient succumbed to septic shock and multiple organ dysfunction syndrome. This is the first case of *Curvularia lunata* infection in our hospital. In this paper, we aim to raise awareness of *Curvularia lunata* infection and to emphasize that the possibility of this fungal infection should be considered in patients with severe pneumonia caused by influenza A virus and that empirical antifungal therapy should be given promptly when the patient has invasive lung damage.

## 1 Introduction

Although *Curvularia lunata* (*C. lunata*) is widely distributed in the environment, human infection is extremely rare. To date, only a few cases of human infection with *C. lunata* have been reported worldwide. Clinical reports have demonstrated that *C. lunata* can lead to various conditions such as dialysis-related peritonitis, fungal keratitis, sinusitis, invasive nasal septal infection, endocarditis, pneumonia, allergic bronchoalveolar disease, mycetoma, brain abscess, skin infections, and disseminated disease (Lopes et al., 1994; Yau et al., 1994; Zheng and Zhao, 2010; Gadgil et al., 2013; Wang et al., 2014). We report here on the case of a young female patient who contracted both bacterial and *C. lunata* infections during influenza A virus infection. Because of the rarity of *C. lunata*, there are currently no standardized diagnostic and treatment guidelines or effective therapies available for this disease. We also compare this case with previously reported cases caused by *C. lunata* to improve the understanding of its clinical manifestations and treatment.

## 2 Case report

**Figure 1:**
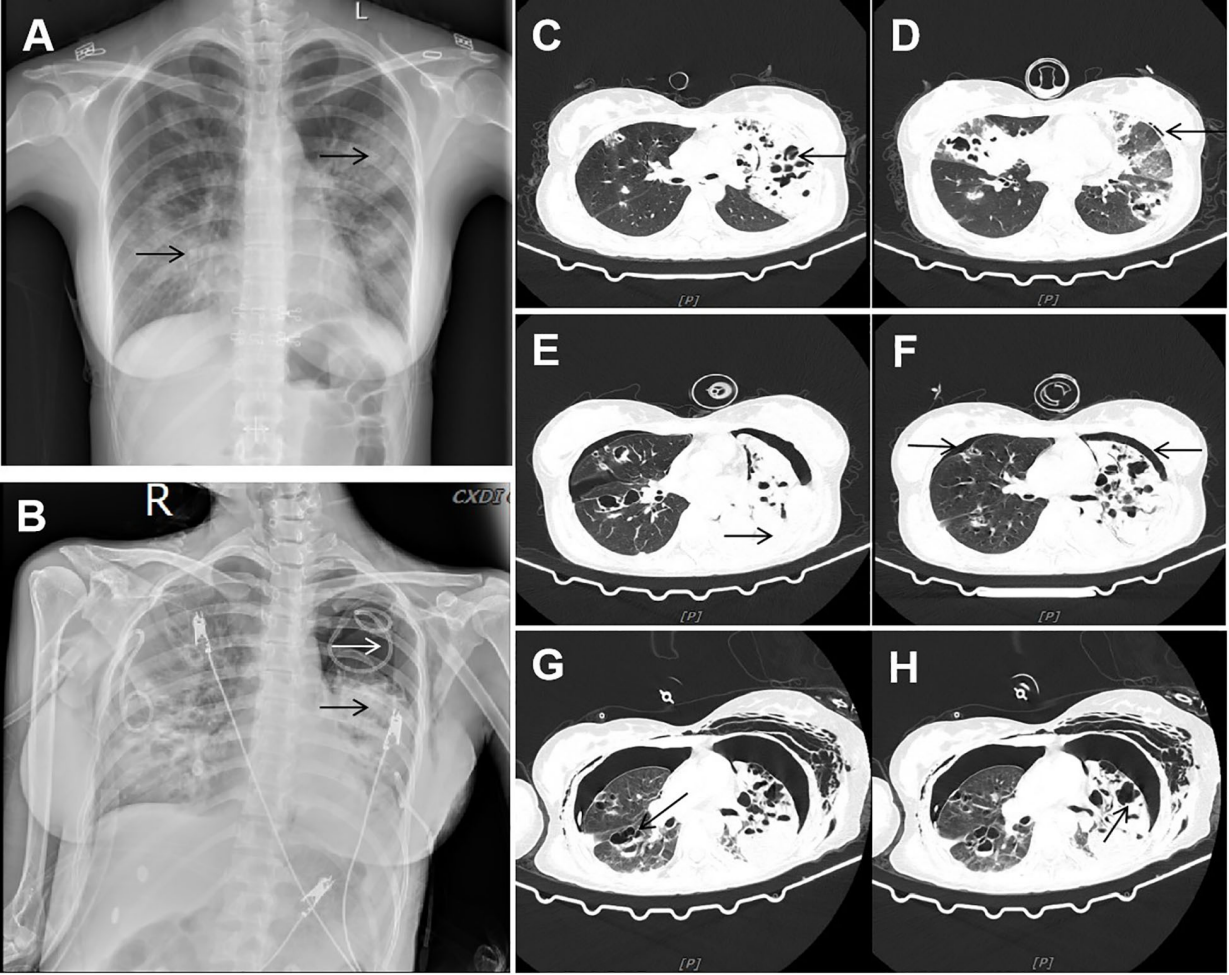
Imaging data. **(A)** Chest X-ray showed multiple patchy and stripe shadows in the lungs. **(B)** Chest X-ray showed that the pneumothorax in the left lung was worse than before, and the left lung was in atelectasis. **(C, D)** CT showed new multiple air cysts in both lungs and new left pneumothorax. **(E, F)** Chest CT showed increased bilateral pleural effusion, enlarged left pneumothorax, and new right pneumothorax. **(G, H)** Chest CT showed large cavities and abscess cavities in both lungs.

The patient, a 23-year-old female, was admitted to the hospital with a one-day history of cough, expectoration, and fever. Her sputum appeared yellow, and a positive result was obtained for the nucleic acid of influenza A virus. Test results showed PCT was 72.8ng/mL, C-reactive protein was 216.15mg/L, neutrophil percentage was 94.9%, WBC was 10.31×10^9^/L, and the chest X-ray displayed the presence of multiple patchy and stripe shadows in both lungs (**Figure 1A**). Severe pneumonia caused by influenza A virus was initially diagnosed. (The treatment timeline for the patient during hospitalization is presented in **Table 1**). Despite a 2-day treatment regimen involving piperacillin sodium, tazobactam sodium antimicrobial, oseltamivir antiviral therapy, atomization, and anticoagulation, the patient’s condition deteriorated with worsening cough and bright red bloody sputum. Furthermore, it became challenging to maintain normal oxygenation levels under mask oxygen inhalation, and multiple organ damage appeared. The clinical manifestations included liver dysfunction, renal dysfunction (Urea/Crea: 159), myocardial damage (myoglobin: 145.4ng/mL), and sputum smear analysis revealed the presence of gram-negative bacilli and gram-positive cocci, indicating a severe bacterial infection. Taking into account the patient’s condition and the sputum smear results, we modified the empirical antibiotic therapy to include a combination of imipenem cilastatin sodium, and linezolid. Simultaneously, the patient received high-flow oxygen therapy and intermittent non-invasive ventilator-assisted respiration while actively collecting respiratory secretion samples for pathogenic microorganism detection. On admission, the patient exhibited low immune indicators, including a lymphocyte percentage of 4.3%, lymphocyte absolute value of 0.44×10^9^/L, IgG level of 7.01g/L, C3 level of 0.55g/L, CD3+ count of 42.9%, and CD3 + CD4+ count of 24.3%. These findings were associated with decreased immune function resulting from influenza A infection. Thymalfasin administration was initiated to improve immune response. Subsequent sputum cultures revealed the presence of *Klebsiella* and *Methicillin-sensitive Staphylococcus aureus (MSSA)*, while respiratory failure showed improvement (switched from high-flow oxygen therapy to nasal cannula oxygen therapy) and the infection index decreased. Consequently, the initial anti-infection treatment plan was maintained.

**Table 1:** The treatment timeline for the patient during hospitalization.

Date	Medications, doses given and frequencies	Clinical condition
14 Mar 2023-15 Mar 2023	Piperacillin Sodium And Tazobactam Sodium (2.5g *bid*)	PCT and C-reactive protein were high. Severe pneumonia caused by influenza A virus was diagnosed.
14 Mar 2023-24 Mar 2023	Oseltamivir (75mg *bid*)	A positive result was obtained for the nucleic acid of influenza A virus.
16 Mar 2023-09 Apr 2023	Imipenem And Cilastatin Sodium (500mg *tid*); Linezolid (0.6g *bid*)	Sputum cultures revealed the presence of *Klebsiella* and *Methicillin-sensitive Staphylococcus aureus (MSSA)*.
21 Mar 2023-30 Mar 2023	Vancomycin (1000mg *bid*)	The *Staphylococcus aureus* infection was not effectively controlled. Intensified the treatment against gram-positive cocci.
30 Mar 2023-04 Apr 2023	Voriconazole (First 24h, 400mg *bid*; 24h after the start of the medication, 200mg, *bid*); Caspofungin (50mg *qd*)	Based on chest CT and fiberoptic bronchoscopy findings, and the presence of mold hyphae in the bronchoalveolar lavage fluid culture, a fungal infection was diagnosed.
05 Apr 2023-09 Apr 2023	Amphotericin B cholesteryl sulfate complex (First 24h, 200mg, *qd*; 24h after the start of the medication, 300mg, *qd*); Isavuconazole (First 48h, 200mg *tid*; 48h after the start of the medication, 200mg, *qd*)	The sputum culture results indicated the presence of *Curvularia lunata*.

The patient was admitted to the hospital on 14 Mar 2023 and passed away on 09 Apr 2023.

(qd, once daily; bid, twice a day; tid, three times a day.).

**Figure 2:**
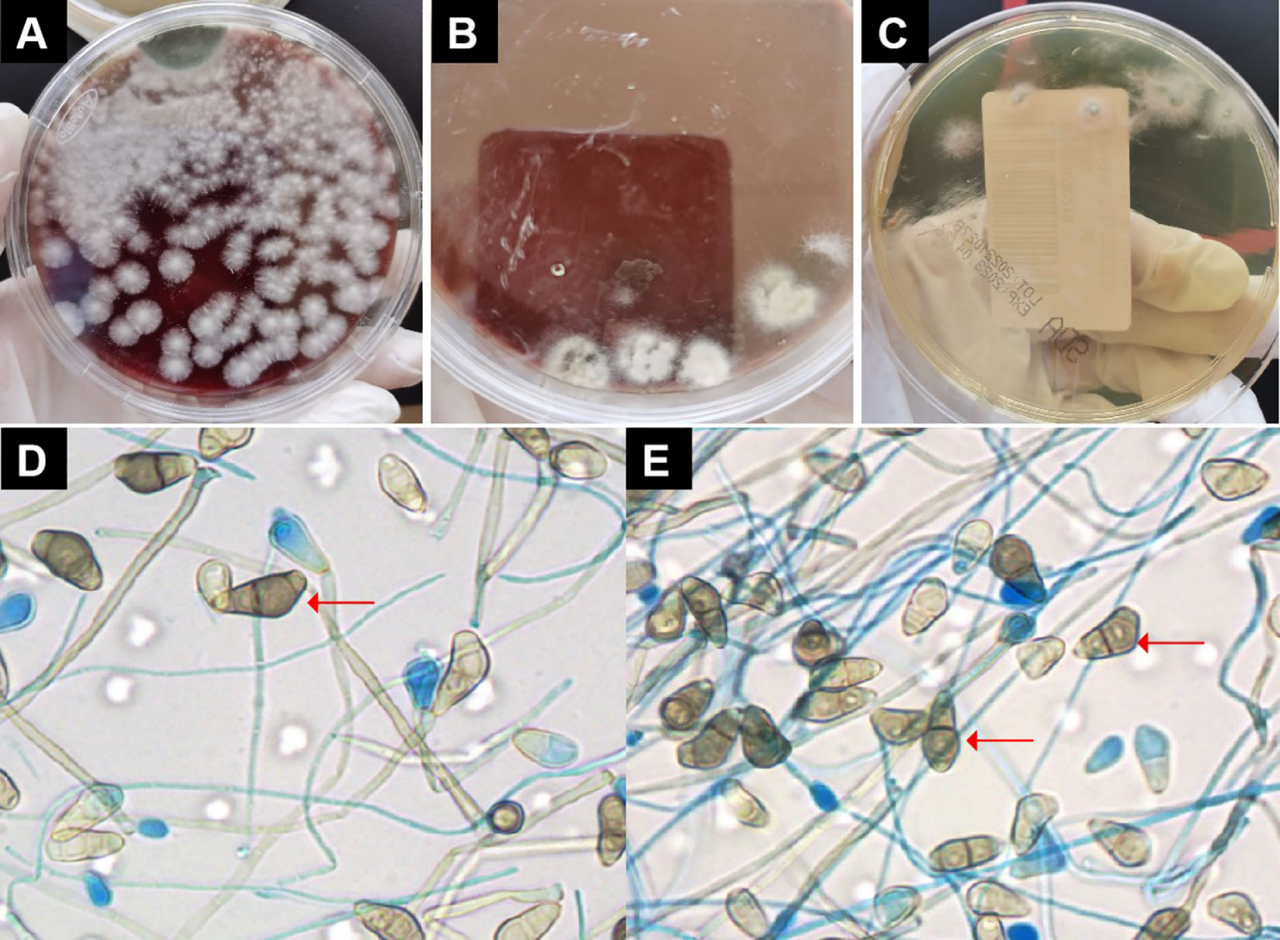
Pathogen culture. **(A)** On blood agar plates could see large numbers of villous or wooly white colonies. **(B)** On chocolate plates could see large numbers of villous or wooly white colonies. **(C)** Colonies on Sabouraud agar plates were seen to be villous, white, with dark olive green in the center and brown on the opposite side. **(D, E)** Under the microscope, the conidiophore was erect and brown in color. The conidia had 3 separated by 4 cells, of which the third cell was curved, darker, and larger. Red arrow indicates *Curvularia lunata.*.

On the eighth day of admission, the patient was conscious but not in good spirits. Reexamination of chest CT showed the presence of newly developed multiple air cysts in both lungs and a new pneumothorax in the left lung (**Figures 1C, D**). Closed thoracic drainage was performed, extracting a significant amount of gas, and purulent and bloody fluid from the drainage tube. The PCT levels continued to decrease, whereas the hemogram showed higher values compared to previous results. Based on the presence of lung abscesses and pneumothorax in the CT scan, we concluded that the *Staphylococcus aureus* infection was not effectively controlled. Therefore, we intensified the treatment against gram-positive cocci by adding vancomycin. On the 11th day of admission, the reexamination of influenza A nucleic acid turned negative, leading to the discontinuation of oseltamivir. Subsequent chest CT revealed an escalation in bilateral pleural effusion, enlargement of the left pneumothorax, and the appearance of a new right pneumothorax (**Figures 1E, F**). Closed thoracic drainage was performed on the right side, from which a significant volume of gas, purulent and bloody fluid was visible. On the thirteenth day of admission, the patient was in poor spirits, and the chest X-ray revealed deterioration of the pneumothorax in the left lung, along with left lung atelectasis (**Figure 1B**). On the seventeenth day of admission, the patient developed numerous pink rashes on her face, neck, chest, and abdomen. As a priority, we considered atopic dermatitis and conducted a screening for potentially allergenic medications. Additionally, the chest CT scan revealed the presence of large cavities and abscess cavities in both lungs (**Figures 1G, H**). Fiberoptic bronchoscopy revealed congestion and edema of the mucosa in both the left and right main bronchi and the lavage fluid exhibited characteristics resembling meat wash. The bronchoalveolar lavage fluid was collected for pathogen culture and inoculated on blood agar plates, chocolate plates, and MacConkey plates. After 24 hours of incubation, needle-like colonies were observed. After 48 hours of incubation, a significant number of fluffy white colonies appeared on blood agar plates (**Figure 2A**) and chocolate plates (**Figure 2B**), with visible growth of mold hyphae under the microscope. Considering both the CT images and the aforementioned findings, a diagnosis of filamentous fungal infection was made. Antifungal therapy was initiated with the addition of voriconazole and caspofungin. It is noteworthy that since the patient’s admission, we conducted multiple G tests and GM tests, all yielding negative results. Additionally, we repeatedly examined bronchodilator vascular fluid and sputum cultures, all of which tested negative except for the presence of fungal hyphae. Subsequently, sputum samples were sent for further fungal culture and inoculated onto Sabouraud agar plates. Following a 24-hour incubation period, colonies on the plates exhibited a villous, white appearance with a dark olive green center and brown on the opposite side (**Figure 2C**). Lactic acid phenol cotton blue staining was conducted, revealing an erect, brown conidiophore under the microscope. The conidia had 3 septa of 4 cells, the third of which was curved, darker in color and larger in size (**Figures 2D, E**). The sputum culture results indicated the presence of *C. lunata*. Immediate antifungal treatment was initiated with amphotericin B cholesteryl sulfate complex and isavuconazole.

On the 24th day of admission, the patient exhibited signs of mental confusion, severe impairment of spirits, ongoing deterioration of her condition, and an escalation of dyspnea (assisted by noninvasive ventilation). The patient experienced gastrointestinal symptoms including gastric retention, gastroparesis, and evident abdominal distension. Gastrointestinal dysfunction resulting from sepsis was taken into consideration, prompting an intensification of gastric motility drugs. Simultaneously, enteral nutrition was decreased and parenteral nutrition was introduced. The patient’s lymphocyte absolute value exhibited a persistent downward trend, necessitating the administration of gamma globulin to enhance her immune response. Considering the patient’s complex condition with difficulties in resolving bilateral pneumothorax, addressing empyema absorption, and persistent atelectasis, a multidisciplinary consultation (MDT) was conducted. It was recommended to replace the drainage tube with a larger diameter to facilitate prompt lung recruitment. Following the replacement, significant quantities of gas and purulent and bloody fluid continued to be drained from the bilateral chest drainage tubes, with chest X-rays indicating a slight re-expansion of the right lung.

On the 25th day, the patient experienced severe cough, chills, high fever, and pronounced dyspnea, with challenging maintenance of oxygen saturation. The patient received emergency treatment involving endotracheal intubation and mechanical ventilation to aid respiration. Under pure oxygen conditions, the blood oxygen saturation level was maintained at 85 - 92%, after which the patient went on to develop shock. The norepinephrine dosage administered was 0.8 μg/kg/min. Electronic bronchoscopy revealed marked airway hyperemia and edema, accompanied by the presence of bloody secretions within the airway. On the 27th day, the patient’s shock continued to worsen without any reduction in ventilator support. The patient was complicated with liver dysfunction, aspartate aminotransferase (AST) was 4283U/L and alanine transaminase (ALT) was 1954U/L, lactic acid increased progressively, and the mouth and nose kept bleeding. The patient’s condition was considered to be aggravated, with poorly controlled infection and secondary disseminated intravascular coagulation. According to the situation, the patient was given an infusion of fresh plasma, prothrombin complex, human fibrinogen, anti-fibrinolytic therapy, and rescue treatments such as rehydration and correction of acidosis. Under the condition of ventilator-assisted respiration and the use of large doses of vasoactive drugs, the patient’s basic vital signs were still difficult to sustain and the patient eventually died.

## 3 Discussion

*Curvularia lunata* belongs to the *Fungi/Fungi Imperfestrainrti/Moniliales/Dematiaceae/Phragmosporoideae/Curvlaria Boedijn* (Zheng and Zhao, 2010). This pathogen has the ability to induce disease in both immunocompetent and immunocompromised hosts and can spread systemically in immunodeficient people. Humans can be infected through respiratory tract or mild skin trauma (Tessari et al., 2003). When the patient was admitted to the hospital, the percentage of lymphocytes was 4.3%, the absolute value of lymphocytes was 0.44×10^9^/L, IgG was 7.01g/L, C3 was 0.55g/L, CD3+ was 42.9%, CD3+ CD4+ was 24.3%, and the immune indicators were low, which was related to the decline of immune function caused by influenza A virus infection. The patient had no prior injuries before admission and no history of contact with suspicious substances. Consequently, we considered that the infection was attributed to a low level of immunity. LeMessurier et al. utilized murine models of acute fungal asthma and influenza to demonstrate that influenza infection could modify the immune response to another significant mold. This finding also gives us a certain inspiration (LeMessurier et al., 2020).

In this case, the chest X-ray showed multiple patchy and striped shadows in both lungs on admission, and the chest CT scan showed bilateral pleural effusion, large exudative and consolidation lesions in both lungs accounting for 60% of the total lung area, which was similar to the CT manifestations of allergic bronchoalveolar disease. Previous research has shown *C. lunata* as a potential cause of allergic bronchoalveolar disease, often linked with elevated eosinophil levels (McAleer et al., 1981). However, the patient’s eosinophil level remained within normal limits, thus excluding the possibility of allergic bronchoalveolar disease. A recent study examining the lungs of mice exposed to the fungal species revealed that the hyphae of *C. lunata* penetrated deeply into the lung parenchyma, leading to interstitial pneumonia and diffuse alveoli characterized by the presence of poorly formed granulomas and multinucleated giant cells. Given the pulmonary invasiveness of *C. lunata*, the possibility that this fungus could spread to other tissues through blood was taken into account (Porter et al., 2011). In this case, the patient developed bloody sputum on the third day after admission, and at a later stage developed large cavities and pus-filled cavities in both lungs and even bilateral pneumothorax, which has not been reported in other cases of *Curvularia* infection. We consider that this was related to the lung invasiveness of *C. lunata*.

In addition to the pulmonary manifestations, the skin manifestations were also typical. Seventeen days after admission, the patient developed numerous pink rashes on the face, neck, chest, and abdomen. Tessari et al. reported a case of fatal systemic dissemination of *Curvularia* infection caused by skin infection in a heart transplant recipient. They claimed that the skin lesions predominantly occurred on the upper and lower arms, with other less common areas like the face, neck, and buttocks. Several days after admission, the heart transplant recipient presented with multiple maculopapular lesions that spread over the skin surface and oral mucosa, displaying shades of pink and brown (Tessari et al., 2003). The findings are generally consistent with the clinical presentation of the patient we reported.

Furthermore, there was one case of localized skin infection due to *C. lunata*: this patient had a local skin infection caused by trauma, and after being injured by a falling tree, a round, ulcerated and crusted lesion formed on his left forearm with almost no secretion and a violaceous, sclerotic border (Hiromoto et al., 2008). Apart from pulmonary and cutaneous infections, *C. lunata* can also cause brain abscess (Gadgil et al., 2013), chronic ambulatory peritoneal dialysis (CAPD) -related infection (Lopes et al., 1994), sinusitis (Yau et al., 1994), invasive nasal septal infection (Sharkey et al., 1990), and keratitis (Wang et al., 2014). Gadgil et al. reported a case of brain abscess attributed to *Curvularia* infection. The patient developed clinical manifestations of dysarthria and ataxia, and a CT scan showed a destructive lesion in the frontal lobe. MRI of the head showed a destructive lesion in the frontal lobe, which was identified by biopsy as *Curvularia* species (Gadgil et al., 2013). When CAPD was accompanied by peritonitis caused by *C. lunata*, the patient was characterized by fever, vomiting, whole abdominal pain and rebound pain, cloudy peritoneal dialysate, and subphrenic abscess on ultrasound examination. The diagnosis required fungal cultures of dialysate and exudate (Lopes et al., 1994). Allergic sinusitis commonly manifested in young adults with a prior history of recurrent rhinitis, asthma, or nasal polyps, accompanied by peripheral eosinophilia and elevated IgG and IgE titers specific for *C. lunata* (Brummund et al., 1986; Yau et al., 1994). When it caused keratitis, the patient had red eyes, eye pain, photophobia, and tears, accompanied by decreased vision. A documented history of corneal trauma aided in diagnosis. However, clinical presentations were frequently atypical, so laboratory etiological examination had great guiding significance for early clinical diagnosis (Wang et al., 2014).

As invasive fungal infections often progress very rapidly, prompt diagnosis and initiation of treatment are essential for patient prognosis. According to the joint clinical guidelines of the European Society for Clinical Microbiology and Infectious Diseases (ESCMID) and the European Confederation of Medical Mycology (ECMM), definitive species identification of phaeohyphomycosis should be performed through histopathology, culture, and sequencing (Chowdhary et al., 2014). Typically, diagnosis necessitates a combination of the patient’s clinical presentation and mycological confirmation. Evidence can be acquired through meticulous microscopic examination, pathology, and fungal culture. Superficial or corneal infections can be diagnosed through direct microscopy without the need for a biopsy. Nevertheless, pathological biopsy remains the gold standard for diagnosing pulmonary and deep localized infections, given that early examination of bronchoalveolar-lavage fluid and drainage fluid often yields negative fungal cultures or smears (He et al., 2022). Throughout the patient’s admission, we had repeatedly reviewed cultures of the bronchoalveolar-lavage fluid and sputum, and the results were negative until the last time we cultured fungi, which was related to stronger bacteria competing with fungi for nutrients and thus inhibiting fungal growth. Besides, we carried out G tests and GM tests a total of three times, until the third time, which was the reexamination after the detection of *Curvularia* species, yielded a positive result, whereas the first two times were negative, suggesting low sensitivity of the G and GM tests for this fungus. Around 2010, matrix-assisted laser desorption ionization time-of-flight mass spectrometry (MALDI-TOF MS) was introduced into the field of clinical microbiology as a more efficient and faster diagnostic technique than DNA sequencing. This technique has been increasingly utilized for mold identification (Lau, 2021). In this case, *C. lunata* was identified at the species level by MALDI-TOF technology.

Since infection with this pathogen is particularly rare, no standardized treatment plan exists worldwide, and conducting randomized clinical trials is infeasible (Revankar and Sutton, 2010). Case reports serve as valuable references to guide treatment planning (Revankar, 2006; Revankar and Sutton, 2010). For visceral or disseminated disease, amphotericin B is preferred, followed by long-term administration of azoles. Studies have suggested that combining azoles with either terbinafine or caspofungin may exhibit synergistic effects (Revankar and Sutton, 2010). Nevertheless, the pathogen has demonstrated resistance to the majority of drugs, and the treatment outcome ultimately relies on the duration of therapy (Vásquez-del-Mercado et al., 2013). For minor local infections such as sinusitis or keratitis, surgery is a helpful option, and it can be combined with systemic antifungal agents (Still et al., 1993; Heinz et al., 1996; Gallelli et al., 2006; Baradkar et al., 2009; Revankar and Sutton, 2010). Hoiromoto et al. demonstrated that terbinafine and itraconazole show effectiveness in treating skin infections (Hiromoto et al., 2008). Gadgil et al. showed that a combination of voriconazole, minocycline, and complete surgical resection results in improved outcomes in patients with brain abscesses (Gadgil et al., 2013).

Wang et al. demonstrated that *C. lunata* is sensitive to itraconazole, voriconazole, and amphotericin B, while resistant to 5-fluorocytosine and fluconazole (Wang et al., 2014). *In vitro* susceptibility studies suggested that treatment with amphotericin B, miconazole, ketoconazole, fluconazole, and itraconazole may be effective (Al-Odaini et al., 2022). A consensus regarding the optimal antifungal treatment for *C. lunata* has not been reached. Although amphotericin B is frequently used for invasive or systemic infections, its success rates are variable (Tessari et al., 2003). Six patients with severe multi-organ infection were treated with amphotericin B and miconazole, but a significant portion experienced poor efficacy or recurrence even after recovery, potentially due to an inadequate dosage of amphotericin B (Lopes et al., 1994). However, there are some cases of successful treatment with amphotericin B. Bryan et al. reported the first successful treatment of endocarditis caused by *Curvularia* infection (Bryan et al., 1993). The disease was effectively controlled after approximately seven years of antifungal treatment, which started with a combination of amphotericin B and ketoconazole, followed by long-term terbinafine treatment. In 1995, a patient with peritoneal dialysis complicated with peritonitis caused by *C. lunata* infection was reported in China. The patient experienced gradual symptom improvement after receiving a total amphotericin B dosage of 500mg. Continuous hemodialysis was administered, leading to the patient’s recovery after six months (Lopes et al., 1994). The efficacy of amphotericin B, with or without oral flucytosine and catheter removal, was demonstrated in the treatment of CAPD-related infections caused by *C. lunata* (Brackett et al., 1988; Ujhelyi et al., 1990; Lopes et al., 1994).

In this case, we started antifungal therapy with isavuconazole and amphotericin B cholesteryl sulfate complex the first time that *C. lunata* was detected. However, because of multiple negative etiologic results during hospitalization, the delay in detection resulted in a relative lag in our treatment timing. Although we started targeted antifungal therapy immediately after the fungus was cultured in the laboratory, the patient had already developed severe lung damage, which contributed to the difficulty in controlling the infection. Moreover, due to the invasion and toxicity of *C. lunata*, as well as the patient’s poor immune function, a variety of factors also contributed to the failure to control the infection. This is also instructive for future cases of such fungal infections: even in the absence of definitive test results, antifungal agents should be employed empirically, particularly in the presence of pertinent clinical symptoms.

There are limitations of our study that are worth mentioning. Although we were able to identify *C. lunata* at the species level using MALDI-TOF MS, the difficulty in detecting this pathogen is indicated by the fact that both bronchoalveolar lavage and sputum cultures, as well as the G test and GM test, were often negative in the early stage. On the one hand, the diagnosis relies on careful microscopic and pathologic examination, as well as clinical evaluation of the patient; on the other hand, there is a need for the development of more effective diagnostic strategies. Moreover, the optimal antifungal treatment for *C. lunata* infections remains to be determined by additional clinical studies.

## 4 Conclusion

Herein, we describe a case of a patient infected with *C. lunata*. This case highlights that negative results from microbiological examinations do not rule out the possibility of invasive fungal infection. Therefore, it is crucial to judge the presence of fungal infection in conjunction with clinical conditions in a comprehensive way. In cases of *C. lunata* infection, prompt empirical treatment should be administered at an early stage when patients exhibit symptoms such as hemoptysis, multiple lung cavities with pus, pneumothorax, or other severe lung damage to avoid missing the best treatment opportunity. Overall, we should direct greater attention towards this rare invasive fungal infection, enhance and optimize diagnostic methods, as well as conduct more in-depth clinical research on these diseases. These efforts will hopefully lead to early diagnosis and more targeted individualized treatment of *C. lunata* infections.

## Data availability statement

The original contributions presented in the study are included in the article/supplementary material. Further inquiries can be directed to the corresponding authors.

## Ethics statement

Ethical approval was not required for the studies on humans in accordance with the local legislation and institutional requirements because only commercially available established cell lines were used. Written informed consent was obtained from the individual(s) for the publication of any potentially identifiable images or data included in this article. Written informed consent was obtained from the participant/patient(s) for the publication of this case report.

## Author contributions

YZ: Writing – original draft, Writing – review & editing. HL: Writing – review & editing. LC: Writing – review & editing. FF: Writing – review & editing. LL: Writing – review & editing. QG: Writing – review & editing.
